# AQP1 Suppresses Clear Cell Renal Cell Carcinoma via Epigenetic Silencing and TNF-Mediated Apoptosis

**DOI:** 10.3390/ijms27125215

**Published:** 2026-06-09

**Authors:** Shuo Pang, Yingwei Bi, Yuxin Liu, Shiming Wang, Bolin Yi, Liang Zhu, Jianbo Wang

**Affiliations:** 1 Department of Urology, The First Affiliated Hospital of Dalian Medical University, Dalian 116011, China; pangshuo0723@163.com (S.P.); biyingwei_@outlook.com (Y.B.); 19845733936@163.com (Y.L.); wsm11070902@163.com (S.W.); yibl@dmu.edu.cn (B.Y.); 2 Advanced Institute for Medical Sciences, Dalian Medical University, Dalian 116041, China

**Keywords:** Aquaporin-1, clear cell renal cell carcinoma, prognosis, epigenetic silencing, TNF signaling

## Abstract

Clear cell renal cell carcinoma (ccRCC) is notorious for its clinical unpredictability. While Aquaporin-1 (AQP1) is a major water channel in healthy kidneys, its specific role and regulatory mechanisms in ccRCC remain unclear. Using bioinformatics analysis of 610 TCGA-KIRC patients (RNA sequencing and DNA methylation), single-cell transcriptomics of 27,402 cells, and experimental validation (CCK-8, scratch, Transwell, and xenograft assays, with Western blotting, HE staining, and immunohistochemistry), we systematically characterized AQP1 expression, regulation, and function. AQP1 was significantly downregulated in ccRCC via promoter hypermethylation, with single-cell analysis confirming tumor cell-specific loss. Low AQP1 correlated with worse prognosis; multivariate Cox regression identified AQP1 as an independent protective factor (HR = 0.510, *p* < 0.001), and a prognostic nomogram showed good predictive accuracy for 1-, 3-, and 5-year survival. AQP1 overexpression suppressed proliferation, migration, invasion, and xenograft growth, accompanied by upregulation of TNF-α, TNFRSF1A, Bax, and Cleaved Caspase-3 and reduced Vimentin, suggesting activation of TNF-related pro-apoptotic signaling. AQP1 is epigenetically silenced in ccRCC and suppresses tumor growth via TNF-mediated apoptosis, establishing it as an independent prognostic biomarker and candidate therapeutic target.

## 1. Introduction

Renal cell carcinoma (RCC) poses a major public health challenge globally. According to 2022 statistics, there were approximately 434,840 new cases worldwide, with the incidence rate climbing by about 1% each year [1]. Within the different histological types, clear cell renal cell carcinoma (ccRCC) appears as the most frequent and aggressive form. It accounts for roughly 75–80% of all cases and causes the majority of RCC-related mortalities [2]. Although modern abdominal imaging has improved our ability to detect incidental renal masses early, managing metastatic progression remains difficult. Data indicates that 25–30% of patients already have synchronous metastases at diagnosis, and a large number of patients with localized disease still experience recurrence even after curative nephrectomy [3,4]. At present, clinicians rely mainly on the Tumor-Node-Metastasis (TNM) staging system for decision-making [5]. However, this anatomical classification has limitations; it often fails to account for the molecular diversity and biological behavior of renal tumors. As a result, patients with the exact same pathological stage can have widely divergent clinical outcomes [6,7]. Therefore, finding robust molecular biomarkers to complement traditional staging is urgent to improve risk stratification and guide personalized therapy.

Aquaporins (AQPs) are a group of small membrane proteins that act as channels, allowing water and solutes to pass through cell membranes rapidly. This discovery fundamentally changed our view of how cells manage fluid [8]. Of the mammalian types, Aquaporin-1 (AQP1) is the most abundant. It is found constantly in the proximal tubule and the thin descending limb of Henle’s loop in the kidney [9]. In terms of physiology, AQP1 is vital. It reabsorbs roughly 80% of the filtered fluid, which helps maintain the balance between glomerular filtration and tubular reabsorption, as well as controlling cell volume [10]. We can see how critical this protein is by looking at Aqp1-knockout mice. These mice lose the ability to concentrate urine (polyuria) and struggle to handle osmotic stress [11]. Interestingly, AQP1 does more than just transport water. Recent evidence points to it affecting other cellular functions, such as cell migration and the growth of new blood vessels (angiogenesis). This suggests it could be involved in disease processes that go beyond simple fluid imbalance.

Problems with AQP1 regulation are linked to various kidney diseases, including acute injury and cancer. Because of this, clinically, it is seen as a potential indicator [12]. For renal cell carcinoma specifically, researchers are focusing on AQP1 as a non-invasive way to detect tumors early. Morrissey and colleagues found that AQP1 levels in urine—often tested alongside Perilipin-2 (PLIN2)—are significantly higher in patients with clear cell or papillary RCC than in healthy people or those with benign conditions [13]. Larger studies have confirmed this, showing that testing for AQP1 has high sensitivity and specificity for finding asymptomatic renal masses [14]. Essentially, tumor cells seem to shed AQP1 into the urine, which makes it an effective target for screening [15]. However, using it as a diagnostic marker in urine is one thing; understanding its biological role inside the tumor is another. We still need to clarify exactly how abnormal AQP1 expression affects the tumor microenvironment and what it predicts for a patient’s long-term survival.

Recent studies indicate that AQP1 behaves differently in cancer depending on the tissue context. While we know AQP1 is vital for water transport in healthy kidneys, its specific role in ccRCC—how it is regulated and what it means for patient outcomes—is still largely a mystery.

## 2. Results

### 2.1. AQP1 Downregulation in ccRCC Correlates with Disease Progression

**Figure 1:**
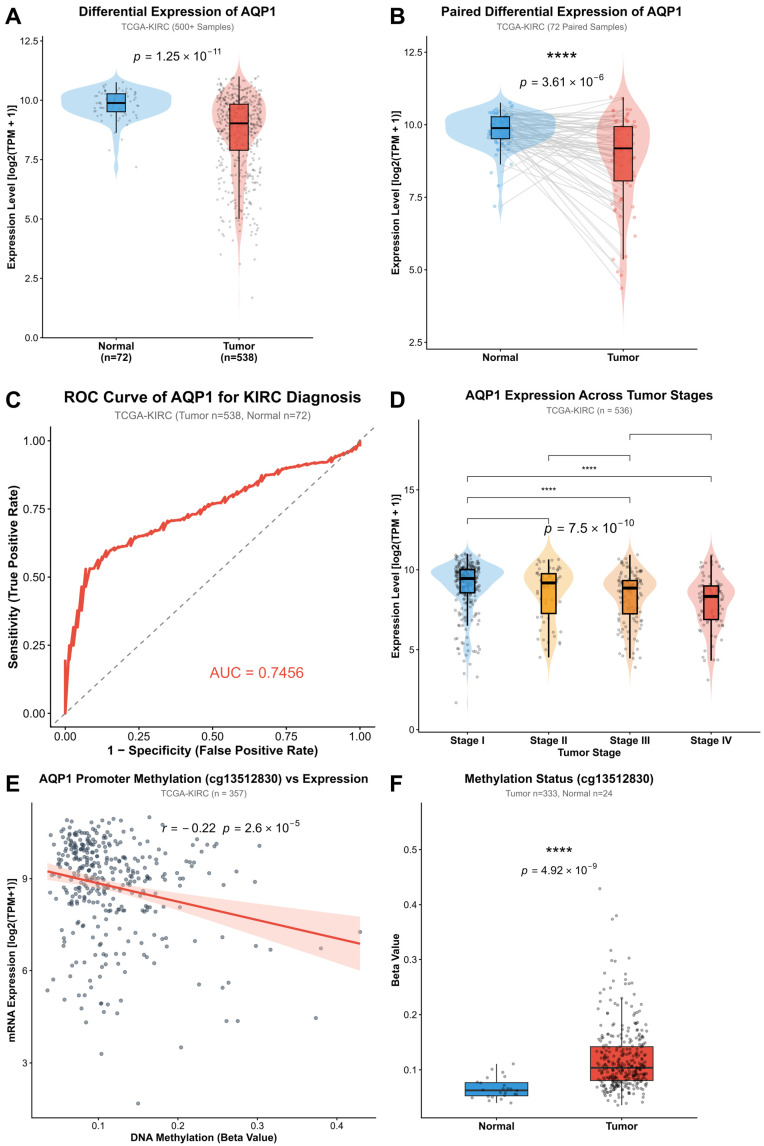
AQP1 expression analysis in clear cell renal cell carcinoma (ccRCC). (**A**) Differential expression of AQP1 in TCGA-KIRC dataset showing significantly lower expression in tumor tissues compared to normal tissues (*n* = 72 normal, *n* = 538 tumor). (**B**) Paired differential expression analysis of AQP1 in TCGA-KIRC paired samples (*n* = 72 paired samples; **** *p* < 0.0001). (**C**) Diagnostic performance of AQP1 showing ROC curve with AUC = 0.7456, indicating moderate diagnostic accuracy for distinguishing tumor from normal tissue. (**D**) AQP1 expression across different tumor stages in TCGA-KIRC cohort (*n* = 536), demonstrating significant differences between Stage I and other stages (**** *p* < 0.0001). (**E**) Correlation analysis between AQP1 expression and DNA methylation levels showing negative correlation (R = −0.22, *p* = 2.6 × 10^−5^), suggesting epigenetic regulation through promoter methylation silencing. (**F**) Methylation status comparison between normal and tumor tissues (**** *p* < 0.0001), with tumor tissues showing higher methylation levels.

Analysis of 610 TCGA-KIRC samples (538 tumor, 72 normal) revealed significant AQP1 downregulation in tumors versus normal kidney tissues in both unpaired (Wilcoxon test, *p* < 0.001; Figure 1A) and paired analyses of 72 matched pairs (paired Wilcoxon test, *p* < 0.001; Figure 1B). ROC curve analysis yielded an AUC of 0.7456 (*p* < 0.001), indicating fair discriminatory ability. While this suggests AQP1 may serve as a supplementary indicator rather than a standalone diagnostic biomarker, its prognostic value was further supported by multivariate Cox regression and nomogram integration with established clinical variables. ROC curve analysis demonstrated acceptable diagnostic performance (Figure 1C).

Stage-stratified analysis revealed progressive AQP1 decline with advancing disease: mean expression decreased from 725.3 TPM in Stage I (n = 271) to 599.9 TPM in Stage II (n = 58), 489.4 TPM in Stage III (n = 123), and 415.2 TPM in Stage IV (n = 84). The Kruskal–Wallis test confirmed significant inter-stage differences (*p* < 0.001), with post hoc pairwise comparisons showing Stage IV expression significantly lower than Stage I (adjusted *p* < 0.001) and Stage II (adjusted *p* < 0.01) (Figure 1D), indicating correlation with tumor progression.

### 2.2. Promoter Hypermethylation Contributes to AQP1 Silencing

Examination of AQP1 promoter methylation using the cg13512830 probe revealed significantly elevated methylation in tumors versus normal tissues (Wilcoxon test, *p* < 0.001; Figure 1F). Correlation analysis demonstrated a significant inverse relationship between promoter methylation and mRNA expression (Pearson’s *r* = −0.22, *p* = 2.5 × 10^−5^; Figure 1E). Although the correlation was modest, this finding suggests promoter hypermethylation represents one mechanism contributing to AQP1 transcriptional repression in ccRCC, likely operating alongside other regulatory pathways.

### 2.3. AQP1 Expression Is an Independent Prognostic Factor in ccRCC

**Figure 2:**
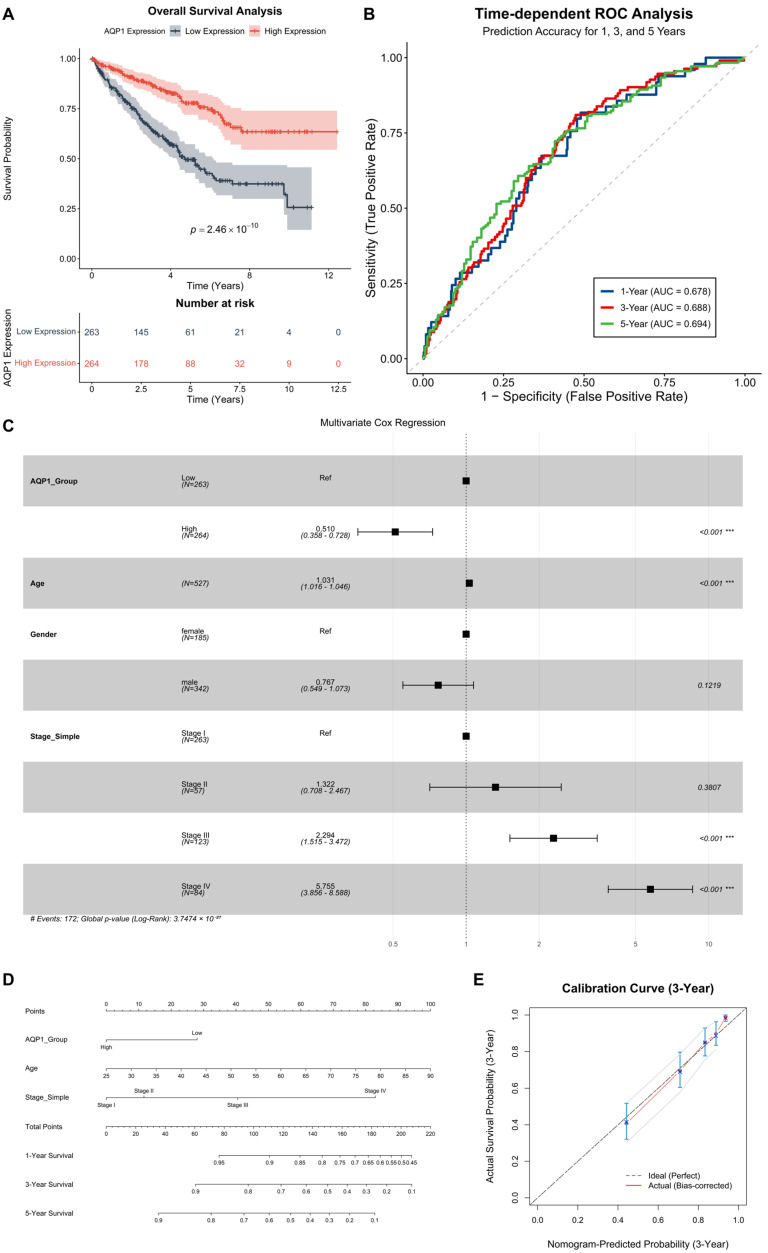
Survival analysis and prognostic prediction model for AQP1 in ccRCC (**A**) Overall survival analysis stratified by AQP1 expression levels. Kaplan–Meier curves show that high AQP1 expression is associated with better prognosis (*p* < 0.001). Risk tables display number of patients at risk at different time points. (**B**) Time-dependent ROC analysis for 1-, 3-, and 5-year survival prediction showing AUC values of 0.678, 0.688, and 0.694, respectively, indicating good predictive accuracy. (**C**) Forest plot of multivariate Cox regression analysis demonstrating AQP1 as an independent prognostic factor along with age, gender, and tumor stage. Hazard ratios with 95% confidence intervals are shown (*** *p* < 0.001). (**D**) Nomogram for predicting 1-, 3-, and 5-year overall survival probability based on AQP1 expression, age, and tumor stage. (**E**) Calibration curve for 3-year survival prediction showing good agreement between nomogram-predicted and actual survival probabilities (ideal vs. actual lines).

Among 527 ccRCC patients with complete clinical data, patients were divided into AQP1-high (*n* = 264; mean: 979.77 TPM) and AQP1-low (*n* = 263; mean: 243.92 TPM) groups using the median expression (525.56 TPM) as the cutoff, representing a 4.02-fold difference. Kaplan–Meier analysis revealed significantly shorter overall survival in the AQP1-low group compared to high expressors (log-rank test, *χ*^2^ = 40.06, *p* < 0.001; Figure 2A). Time-dependent ROC analysis demonstrated moderate prognostic accuracy at 1-year (AUC = 0.678), 3-year (AUC = 0.688), and 5-year (AUC = 0.694)(Figure 2B). Although these values indicate fair rather than excellent discrimination, AQP1′s prognostic significance was reinforced by its identification as an independent factor in multivariate Cox regression and its integration into a nomogram with clinical variables.

Multivariate Cox regression adjusting for age, gender, and pathological stage identified high AQP1 expression as an independent protective factor (HR = 0.510, 95% CI: 0.358–0.728, *p* < 0.001; Figure 2C). Other independent prognostic factors included age (HR = 1.031 per year, 95% CI: 1.016–1.046, *p* < 0.001) and advanced stage, with Stage III (HR = 2.294, 95% CI: 1.515–3.472, *p* < 0.001) and particularly Stage IV (HR = 5.755, 95% CI: 3.856–8.588, *p* < 0.001) showing markedly elevated mortality risk compared to Stage I. Gender showed a borderline trend, with males demonstrating marginally better survival than females (HR = 0.767, 95% CI: 0.549–1.073, *p* = 0.122). Stage II exhibited no significant difference from Stage I (HR = 1.322, 95% CI: 0.708–2.467, *p* = 0.381).

A prognostic nomogram integrating AQP1 expression with significant clinical variables was developed to predict individual 1-, 3-, and 5-year survival probabilities (Figure 2D). Calibration curves demonstrated excellent agreement between predicted and observed survival (Figure 2E), validating the model’s clinical utility.

### 2.4. AQP1 Overexpression Activates Inflammatory and Immune Regulatory Pathways

**Figure 3:**
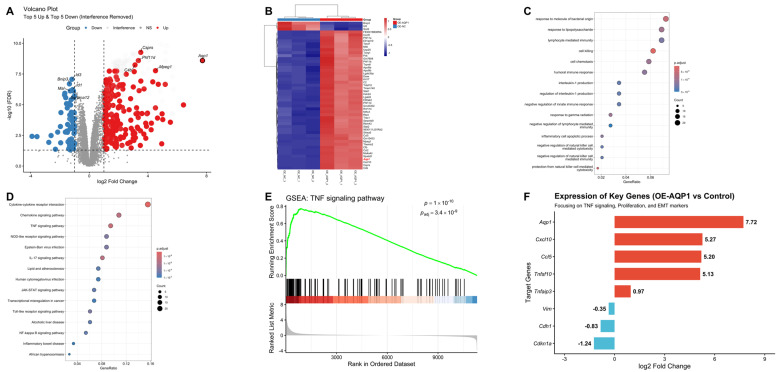
Transcriptomic analysis and pathway enrichment of AQP1-associated genes. (**A**) Volcano plot showing differentially expressed genes between high and low AQP1 expression groups. Top 5 upregulated (red) and downregulated (blue) genes are labeled, with Aqp1 highlighted. (**B**) Heatmap displaying expression patterns of selected genes across samples, clearly distinguishing between oe-NC and oe-AQP1 groups. (**C**) GO biological process enrichment analysis showing significant pathways including lymphocyte mediated immunity, cell killing, chemotaxis, and immune response processes. Dot plot shows gene ratios and adjusted *p*-values. (**D**) KEGG pathway enrichment analysis highlighting TNF signaling pathway, chemokine signaling pathway, cytokine-cytokine receptor interaction, and other immune-related pathways. (**E**) GSEA enrichment plot for TNF signaling pathway (*p* value = 1 × 10^−10^, *p*.adjust = 3.4 × 10^−9^) showing significant positive enrichment. The green curve represents the running enrichment score; black vertical lines indicate the positions of pathway member genes within the ranked gene list; the red-to-blue color bar represents the ranked list metric, with red denoting genes more highly expressed in the OE-AQP1 group and blue denoting genes more highly expressed in the control group. (**F**) Expression levels of key genes focusing on TNF signaling, proliferation, and EMT markers, displaying log2 fold changes for genes including Aqp1, Tnfsf10, Ccl5, Cxcl10, and others. Bar plot of log_2_ fold-change for key genes in OE-AQP1 versus control cells. Red bars indicate upregulated genes (log_2_FC > 0) and blue bars indicate downregulated genes (log_2_FC < 0).

RNA sequencing of AQP1-overexpressing versus control mouse kidney cells identified 283 differentially expressed genes (DEGs) after filtering transfection artifacts (Supplementary Table S1): 220 upregulated and 63 downregulated (|log_2_FC| > 1, FDR < 0.05; Figure 3A). AQP1 was among the most highly upregulated genes (log_2_FC = 7.72, FDR = 2.56 × 10^−9^). Hierarchical clustering of top DEGs clearly separated experimental groups (Figure 3B).

Gene Ontology enrichment revealed significant over-representation of immune-related processes: ‘cell killing’ (FDR = 4.82 × 10^−5^), ‘protection from NK cell-mediated cytotoxicity’ (FDR = 5.59 × 10^−5^), ‘humoral immune response’ (FDR = 2.27 × 10^−4^), and ‘cell chemotaxis’ (FDR = 3.25 × 10^−4^) (Figure 3C). KEGG pathway analysis identified enrichment of key inflammatory cascades: ‘TNF signaling pathway’ (12 genes, adjusted *p* = 3.58 × 10^−6^), ‘Chemokine signaling’ (14 genes, adjusted *p* = 8.47 × 10^−6^), ‘Cytokine-cytokine receptor interaction’ (17 genes, adjusted *p* = 8.47 × 10^−6^), ‘IL-17 signaling’ (10 genes, adjusted *p* = 1.73 × 10^−5^), and ‘NF-κB signaling’ (7 genes, adjusted *p* = 7.67 × 10^−3^) (Figure 3D). The prominent TNF pathway enrichment prompted further investigation of AQP1-TNF interactions in the tumor microenvironment (complete GO results in Supplementary Table S2; KEGG results in Supplementary Table S3).

GSEA further confirmed the transcriptional activation of the TNF signaling pathway at a gene-set level, yielding a significant positive enrichment score (*p*-value = 1 × 10^−10^, adjusted *p* = 3.4 × 10^−9^; Figure 3E). To further characterize the functional implications of AQP1 overexpression, we examined the expression of key genes across three functionally relevant categories (Figure 3F). Among TNF signaling-related genes, Tnfsf10 (log_2_FC = 5.13), Ccl5 (log_2_FC = 5.20), Cxcl10 (log_2_FC = 5.27), and Tnfaip3 (log_2_FC = 0.97) were notably upregulated. In contrast, EMT-associated markers showed a suppressive trend, with Vim (log_2_FC = −0.35) and Cdh1 (log_2_FC = −0.83) downregulated, alongside the cell cycle inhibitor Cdkn1a (log_2_FC = −1.24). These results collectively suggest that AQP1 overexpression not only activates inflammatory signaling but may also suppress tumor cell plasticity and proliferative capacity.

### 2.5. Single-Cell Profiling Reveals Cell Type-Specific AQP1 Distribution

**Figure 4:**
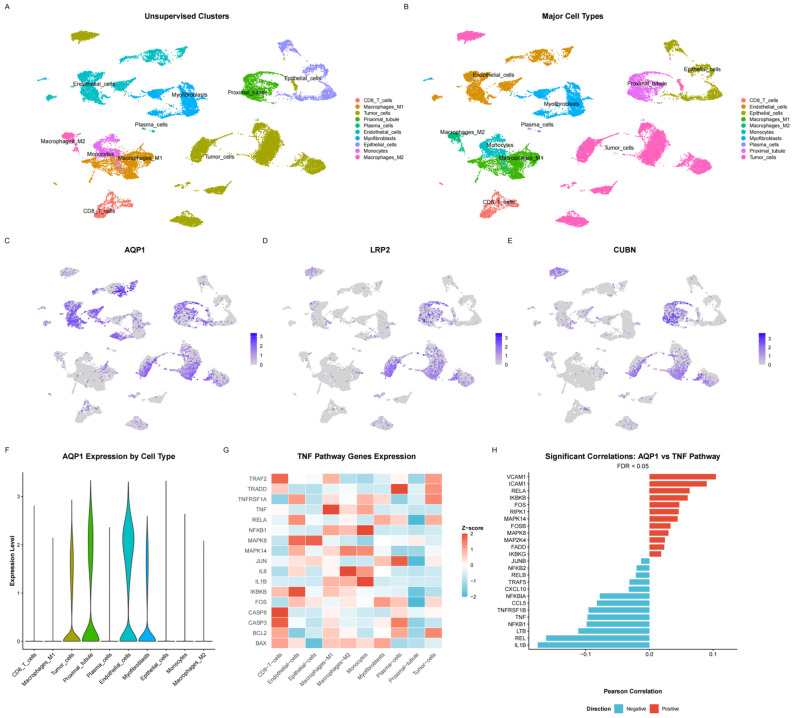
Single-cell RNA sequencing analysis of AQP1 expression in ccRCC. (**A**) UMAP visualization of unsupervised cell clusters identified in the single-cell dataset, showing distinct cell populations. (**B**) Cell type annotation of major cell types including CD8+ T cells, macrophages, tumor cells, proximal tubule cells, plasma cells, endothelial cells, myofibroblasts, epithelial cells, and monocytes. (**C**–**E**) Feature plots showing expression of AQP1 (**C**), LRP2 (**D**), and CUBN (**E**) across different cell types, with expression levels indicated by color intensity. (**F**) Violin plot quantifying AQP1 expression levels across different cell types, showing highest expression in proximal tubule cells and Epithelial cells. (**G**) Heatmap of TNF pathway genes expression across different cell types, displaying Z-scores for genes including BAX, BCL2, CASP3, CASP8, TNF, TNFRSF1A, and others. (**H**) Correlation analysis between AQP1 and TNF pathway genes showing significant correlations (FDR < 0.05) with positive and negative correlations indicated by color coding.

Single-cell analysis of 27,402 ccRCC cells allowed us to identify 10 major cell types via unsupervised clustering (Figure 4A,B). To validate our cell annotations, we checked canonical markers. AQP1 expression was highly specific (Figure 4C) and spatially co-localized with the proximal tubule markers LRP2 (Figure 4D) and CUBN (Figure 4E). Quantitative analysis confirmed this pattern. As shown in the violin plots, AQP1 expression was highest in proximal tubule cells and moderate in epithelial cells, while remaining minimal in immune and stromal populations (Figure 4F). This single-cell resolution validates that the AQP1 loss observed in bulk tissues primarily reflects the downregulation in tumor cells rather than a loss of normal tissue composition.

### 2.6. AQP1 Associates with TNF Signaling in the Tumor Microenvironment

Analysis of 40 core TNF pathway genes (Supplementary Table S4) across cell types revealed heterogeneous pathway activation: high in immune cells (macrophages, monocytes, T cells), moderate in stromal cells (myofibroblasts, endothelial cells), and lower in tumor and proximal tubule cells (Figure 4G). Pearson correlation analysis across all 27,402 cells identified multiple significant AQP1-TNF gene correlations (FDR < 0.05; Figure 4H), with both positive and negative associations detected. These context-dependent relationships suggest AQP1 may differentially modulate TNF-mediated inflammatory responses across distinct cellular niches within the kidney microenvironment, potentially influencing tumor-immune interactions.

Collectively, these multi-scale analyses establish AQP1 as a consistently downregulated gene in ccRCC through epigenetic mechanisms, strongly associated with advanced disease stage and poor prognosis, and functionally linked to inflammatory signaling networks. These findings nominate AQP1 as a promising prognostic biomarker and potential therapeutic target warranting further mechanistic investigation.

### 2.7. Establishment and Verification of AQP1-Overexpressing RENCA Cells

**Figure 5:**
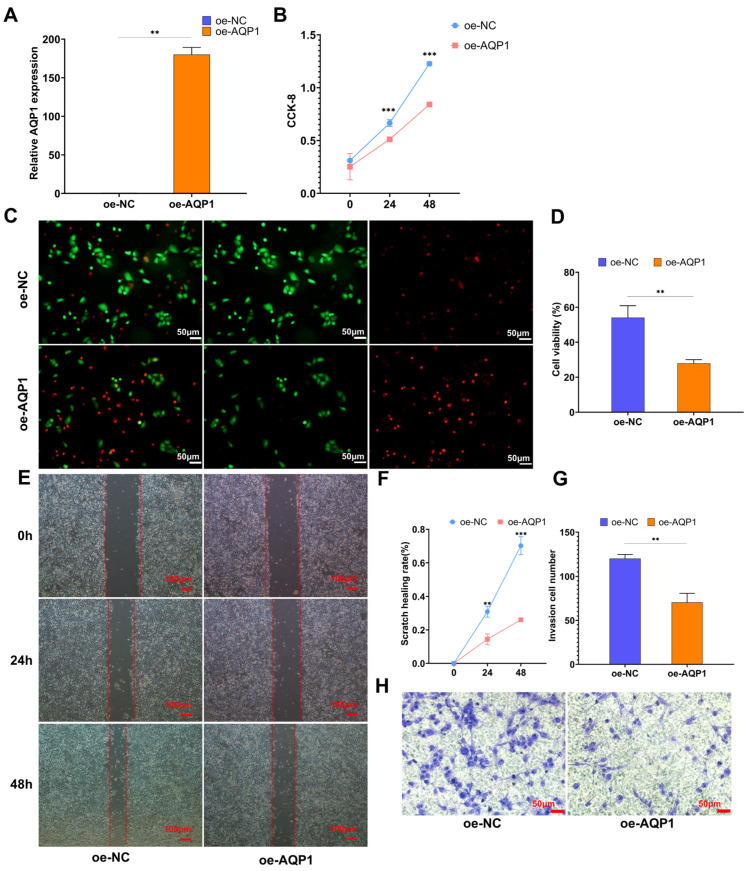
AQP1 overexpression inhibits proliferation, viability, migration, and invasion in vitro. (**A**) Quantitative PCR validation showing successful AQP1 overexpression in oe-AQP1 group compared to oe-NC control. (** *p* < 0.01). (**B**) CCK-8 assay demonstrating significantly suppressed cell proliferation in the oe-AQP1 group at 24 h and 48 h. By 48 h, OD values were markedly lower in the oe-AQP1 group (0.840 ± 0.017) than in the oe-NC group (1.227 ± 0.017) (*** *p* < 0.001). (**C**) Representative fluorescence microscopy images of Calcein-AM/PI staining (live cells in green, dead cells in red; scale bar: 50 μm). (**D**) Quantification of cell viability showing a significant reduction in the oe-AQP1 group (27.97%) compared to oe-NC (54.08%) (*p* = 0.003) (** *p* < 0.01). (**E**) Representative scratch assay images at 0 h, 24 h and 48 h; red dashed lines indicate the scratch wound margins (scale bar: 100 μm). (**F**) Quantification of scratch healing rate over time, showing significantly lower migration in the oe-AQP1 group versus oe-NC control at 24 h (14.44 ± 3.17% vs. 30.89 ± 3.25%, ** *p* = 0.0033) and 48 h (26.02 ± 0.94% vs. 70.21 ± 5.37%, *** *p* < 0.001). (**G**) Quantification of invaded cell number showing significant reduction in the oe-AQP1 group (70.33 ± 10.41) compared to control (120 ± 4.58) (*p* = 0.002) (** *p* < 0.01). (**H**) Representative Transwell invasion images (scale bar: 50 μm). All in vitro data are presented as the mean ± SD from three independent experiments (n = 3); *p* values were calculated by two-tailed unpaired Student’s *t*-test. ** *p* < 0.01, *** *p* < 0.001.

To investigate the biological role of AQP1 in renal cell carcinoma, we established a stable AQP1-overexpressing RENCA cell line using lentiviral transduction. qPCR analysis confirmed that the mRNA level of AQP1 was significantly increased by approximately 163.4-fold in the oe-AQP1 group compared with the oe-NC group (*p* < 0.001). This result successfully validated the efficiency of the overexpression system for subsequent experiments (Figure 5A).

### 2.8. AQP1 Overexpression Inhibits Proliferation, Viability, Migration, and Invasion In Vitro

Cell Proliferation (CCK-8): The CCK-8 assay demonstrated that AQP1 significantly suppressed cell proliferation. A significant divergence between the two groups was observed starting from 24 h (*p* < 0.001). By 48 h, the OD value of the oe-AQP1 group (0.840 ± 0.017) was markedly lower than that of the oe-NC group (1.227 ± 0.017) (Figure 5B).

Cell Viability (Calcein-AM/PI): Calcein-AM/PI staining revealed that AQP1 overexpression significantly reduced cell viability (Figure 5C). The live cell rate dropped from 54.08% in the oe-NC group to 27.97% in the oe-AQP1 group (*p* = 0.003) (Figure 5D).

Migration and Invasion: The scratch assay showed that the scratch healing rate in the oe-AQP1 group was significantly lower than that in the oe-NC group at both 24 h (14.44 ± 3.17% vs. 30.89 ± 3.25%, *p* = 0.0033) and 48 h (26.02 ± 0.94% vs. 70.21 ± 5.37%, *p* < 0.001) (Figure 5E,F). Additionally, the Transwell assay indicated that the number of invaded cells decreased from 120 ± 4.58 in the control group to 70.33 ± 10.41 in the oe-AQP1 group (*p* = 0.002) (Figure 5G,H).

### 2.9. AQP1 Significantly Inhibits Tumor Growth In Vivo

**Figure 6:**
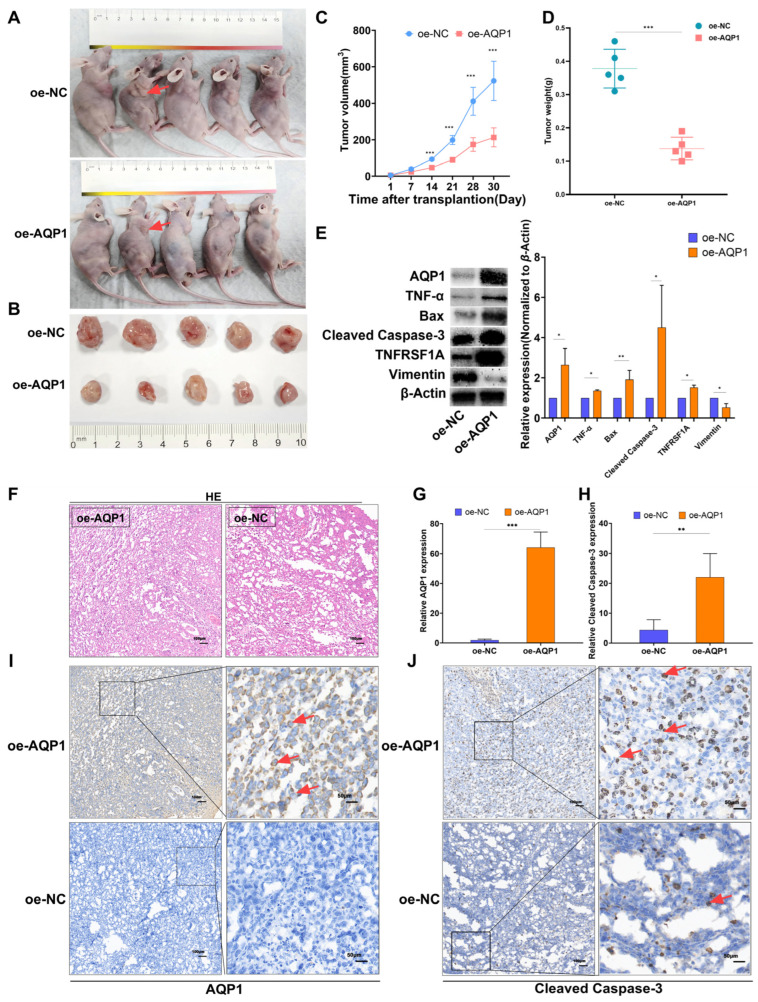
In vivo validation of AQP1 function using xenograft mouse model. (**A**) Representative images of nude mice bearing subcutaneous tumors from oe-NC control and oe-AQP1 groups, showing visible tumor size differences (red arrows indicate subcutaneous tumors). (**B**) Tumor specimen images showing size comparison between groups, with measurements indicated by ruler scale (0–10 cm). (**C**) Tumor volume growth curves over 30 days post-transplantation, demonstrating significantly inhibited tumor growth in the oe-AQP1 group compared to the oe-NC control. By day 30, the mean tumor volume in the oe-AQP1 group (212.88 ± 46.55 mm^3^) was markedly reduced relative to the oe-NC group (522.81 ± 96.45 mm^3^) (*** *p* < 0.001). (**D**) Final tumor weight at day 30 showing significantly reduced tumor weight in the oe-AQP1 group (0.138 ± 0.034 g) compared to the oe-NC control group (0.378 ± 0.058 g) (*** *p* < 0.001), consistent with the observed suppression of tumor growth. (**E**) Western blot analysis of tumor tissues probing for AQP1, TNF-α, Bax, Cleaved Caspase-3, TNFRSF1A, Vimentin, and β-Actin (loading control), with oe-NC and oe-AQP1 groups comparison (** *p* < 0.01, * *p* < 0.05). (**F**) Hematoxylin and Eosin (HE) staining of tumor sections, showing increased structural damage and necrotic areas in the oe-AQP1 group (Scale bar = 100 μm). (**G**) Quantitative analysis of AQP1 immunohistochemical (IHC) staining intensity in tumor sections, showing a significant increase in AQP1 expression in the oe-AQP1 group (*** *p* < 0.001). (**H**) Quantitative analysis of Cleaved Caspase-3 IHC staining intensity, demonstrating enhanced apoptotic activity in AQP1-overexpressing tumors (** *p* < 0.01). (**I**) Representative IHC images of AQP1 in tumor tissues from oe-NC and oe-AQP1 groups. **Left panels**: low magnification (scale bar = 100 μm); **Right panels**: high magnification of the boxed areas (scale bar = 50 μm); red arrows indicate representative AQP1-positive (DAB+) cells. (**J**) Representative IHC images of Cleaved Caspase-3 staining, illustrating the spatial distribution of apoptotic cells in tumor sections. **Left panels**: low magnification (scale bar = 100 μm); **Right panels**: high magnification (scale bar = 50 μm); red arrows indicate representative Cleaved Caspase-3-positive (apoptotic) cells. Animal data are presented as the mean ± SD (n = 5 mice per group); *p* values were calculated by two-tailed unpaired Student’s *t*-test, * *p* < 0.05, ** *p* < 0.01, *** *p* < 0.001.

The anti-tumor effect of AQP1 was further evaluated using a nude mouse xenograft model (Figure 6A,B):

1. Tumor Volume: The growth curves showed that the tumors in the oe-AQP1 group grew significantly slower than those in the control group. By day 30, the average tumor volume in the oe-AQP1 group (212.88 ± 46.55 mm^3^) was significantly reduced compared with the oe-NC group (522.81 ± 96.45 mm^3^) (*p* < 0.001) (Figure 6C) (Table 1).
2. Tumor Weight: At the end of the experiment (day 30), the average tumor weight in the oe-AQP1 group was significantly lower than that in the control group (0.138 ± 0.034 g vs. 0.378 ± 0.058 g, *p* < 0.001) (Figure 6D) (Table 1).

### 2.10. AQP1 Overexpression Is Associated with TNFα Pathway Modulation and Increased Apoptotic Markers In Vivo

To explore the potential mechanisms underlying AQP1′s anti-tumor effects, we evaluated histological changes and protein expression in xenograft tissues. Western blot analysis confirmed sustained AQP1 protein expression in the oe-AQP1 group. Notably, AQP1 overexpression was associated with a significant elevation in TNFα and its receptor TNFRSF1A levels, suggesting a potential involvement of inflammatory signaling in AQP1-mediated tumor suppression. Furthermore, markers of apoptosis were significantly modulated. The pro-apoptotic protein Bax and the cleaved form of Caspase-3 were upregulated in AQP1-overexpressing tumors. We also observed a reduction in the mesenchymal marker Vimentin, which may indicate an inhibitory effect on tumor cell plasticity (*p* < 0.05, Figure 6E). Histological examination via HE staining showed that tumors in the oe-AQP1 group exhibited more pronounced structural alterations and necrotic areas compared to the control group (Figure 6F). These observations were further supported by IHC staining, showing increased immunoreactivity for Cleaved Caspase-3 in AQP1-overexpressing tissues (Figure 6G–J). Taken together, these findings suggest that AQP1 may suppress tumor growth by fostering a pro-apoptotic microenvironment, a process potentially involving the modulation of the TNFα/TNFRSF1A signaling axis.

## 3. Discussion

It seems that Aquaporin-1 (AQP1) plays a dual role in cancer, acting differently depending on the specific tissue. In our study on ccRCC, we found it acts as a tumor suppressor and is downregulated. This stands in sharp contrast to other malignancies where AQP1 is often upregulated and pushes tumor progression. For example, in gastric cancer, high AQP1 levels help cells grow and invade by activating the GRB7/ERK/Ras signaling axis [16]. It also connects to VEGF expression and microvessel density, promoting angiogenesis [17,18]. The story is similar in lung adenocarcinoma. There, high AQP1 is a recognized risk factor for poor prognosis and is frequently linked to aggressive clinical features [19,20].

The situation in colorectal carcinoma is complicated. Some research suggests AQP1 helps predict how well chemotherapy works [21]. However, a key study by Smith et al. found that AQP1 levels are actually lower in tumor tissues because of promoter hypermethylation [22]. These data are consistent with our research. It implies that in some cancers, turning off AQP1—and disrupting the cell’s water balance—is a critical step in starting the tumor. This points to a dual role for the protein: in some places, high AQP1 helps cancer cells change shape and spread. But in the kidney, losing AQP1 seems to trigger stress responses, specifically the TNF pathway we identified.

At a molecular level, scientists now view ccRCC as a metabolic disease because it completely changes how cells use energy [23]. AQP1 is key for moving water and regulating pressure. So, when tumor cells shut it down, it is likely a survival strategy to adapt to this new metabolic environment. Our survival data matches recent large-scale clinical findings. For example, Lee et al. looked at 827 ccRCC cases using immunohistochemistry. They confirmed that losing AQP1 protein links directly to worse TNM stages and shorter survival [24]. Independent studies looking at gene expression across multiple groups back this up [25]. This makes AQP1 a reliable predictor of prognosis.

But relying on just one biomarker is rarely enough because every patient is different. Recently, researchers proposed complex risk models using stemness-related lncRNA signatures or single-cell data [26,27]. While interesting, these methods are often expensive and hard to analyze, making them difficult to use in daily practice. Instead, the nomogram we built combines AQP1 levels with standard clinical data. This offers a tool that is both cheap and practical for assessing risk. Our combined model predicts long-term survival better than single-gene markers like ERBB2 [28]. Similar nomograms have successfully predicted lung metastasis and survival in other kidney cancers [29,30]. This suggests that adding AQP1 to these quantitative models is a smart way to personalize cancer treatment.

Our study revealed that AQP1 restoration is associated with activation of the TNF signaling pathway, pointing to a possible pro-apoptotic mechanism that remains to be functionally validated. At first, this seems contradictory because TNF-α is usually known for causing inflammation and helping tumors grow through chronic NF-κB activation [31,32]. But TNF is actually a “double-edged sword” in cancer [33]. Whether a cell lives or dies depends entirely on the specific context and signal thresholds. The outcome of TNFR1 signaling depends on which adaptor proteins are recruited. Normally, TRAF2 joins with cIAP1/2 to ubiquitinate RIPK1, which sends out survival signals [34,35]. However, if the cytotoxicity threshold is crossed—as Vredevoogd et al. showed—the signaling shifts. It forms a death-inducing signaling complex (DISC) that recruits FADD and Caspase-8 to kill the cell [36]. Our Western blot data supports this. We saw high levels of Bax and Cleaved Caspase-3, suggesting that overexpressing AQP1 disrupts the balance of fluids or metabolism in ccRCC cells. This was further corroborated at the tissue level, where HE staining revealed pronounced necrotic areas and IHC confirmed elevated Cleaved Caspase-3 immunoreactivity in AQP1-overexpressing xenografts. We hypothesize that restoring AQP1 may shift TNFR1 signaling toward a pro-apoptotic output, a possibility that requires direct functional testing. We see something similar in lung adenocarcinoma, where certain regulators like TRAF3IP3 use ER stress to force tumor cells into apoptosis [37]. Essentially, restoring AQP1 is not just a structural fix; it functions as a “re-sensitizer” to cell death.

When we looked upstream to find why AQP1 is lost, we identified promoter hypermethylation as the main culprit. This supports the growing view that ccRCC is essentially an “epigenetic disease,” shaped by broad DNA methylation changes that go well beyond the classic VHL mutations [38]. Just as the methylation of QPCT drives resistance to sunitinib [39], the epigenetic silencing of AQP1 seems to be another key step in how ccRCC progresses. Recent single-cell multi-omics studies (mixing scRNA-seq and scATAC-seq) shed light on this heterogeneity. They showed that the differences between ccRCC cells are largely controlled by how open the chromatin is and their methylation status [40]. This likely explains the variation in AQP1 levels we saw within tumors.

Because epigenetic silencing is, in principle, reversible, our data raise the possibility that restoring AQP1 could have therapeutic value. DNA methyltransferase inhibitors can de-repress silenced tumor-suppressor genes [41], and more targeted dCas9-based epigenetic editors—programmable activators (e.g., TET1–dCas9) delivered transiently as ribonucleoprotein complexes—can in principle reactivate a single silenced locus without permanently altering the genome [42]. We emphasize, however, that these ideas remain hypothetical for ccRCC and face two major obstacles. First, efficient and selective in vivo delivery of such editors to renal cancer cells is still unsolved. Second, because AQP1 is oncogenic in several other tumor types, including gastric and lung cancers [16,17], any reactivation strategy would require tumor- or cell-type-restricted targeting to avoid promoting tumor growth elsewhere. These therapeutic directions are therefore presented as hypotheses generated by, and not conclusions of, the present study. Consequently, the priorities emerging from our findings are primarily mechanistic. Future studies should directly test whether AQP1 restoration induces apoptosis in vivo (e.g., by TUNEL on tumor sections), determine whether this effect is genuinely TNF-dependent using TNF/TNFR1 blockade or genetic perturbation, incorporate a parental (non-transduced) control together with human ccRCC and patient-derived models, and assess in clinical cohorts whether AQP1 status predicts response to immune checkpoint inhibitors before any predictive-biomarker role is claimed. Although immune checkpoint inhibitors are now central to the treatment of metastatic ccRCC, primary and acquired resistance remain common [43,44,45], and effective anti-PD-1 activity requires not only T-cell infiltration but also competent T-cell-mediated tumor killing [46]; whether AQP1 loss contributes to immune escape by raising the apoptotic threshold is an intriguing but unproven possibility that warrants dedicated investigation.

## 4. Materials and Methods

### 4.1. Data Acquisition and Preprocessing

RNA sequencing data for ccRCC were obtained from TCGA via the GDC Data Portal. After quality control, 610 samples (538 tumor, 72 normal) were retained. Gene expression was quantified as TPM and log_2_-transformed [log_2_(TPM + 1)]. DNA methylation data from the same cohort (Illumina HumanMethylation450) were used to assess AQP1 promoter methylation (TSS200 region) via beta values. Single-cell RNA sequencing data were acquired from GEO (GSE159115). In vitro transcriptome data were generated from AQP1-overexpressing RENCA cells (n = 3) versus negative controls (n = 3), sequenced by Majorbio Bio-pharm Technology (Shanghai, China) and aligned to the GRCm39 reference genome. Sample sizes are summarized in Table 2.

### 4.2. Differential Expression and Clinical Association Analysis

AQP1 mRNA expression was compared between tumor and normal samples using Wilcoxon tests (unpaired and paired). Stage-stratified differences were assessed by Kruskal–Wallis test with pairwise BH-corrected comparisons (FDR < 0.05). ROC analysis was performed to evaluate diagnostic performance (AUC with 95% CI; optimal cutoff by Youden’s index). Methylation-expression correlation was assessed by Pearson correlation. All tests were two-sided (α = 0.05).

### 4.3. Prognostic Analysis and Nomogram Development

Clinical metadata were integrated with expression data; samples with missing survival information were excluded, retaining 527 tumor samples. Patients were stratified into AQP1-high and AQP1-low groups using the median expression value (525.56TPM; log2-transformed: 9.04) as the cutoff, resulting in two balanced groups (AQP1-high n = 264 and AQP1-low n =263), and Kaplan–Meier curves were compared by log-rank test. Time-dependent ROC curves were constructed for 1-, 3-, and 5-year overall survival. Multivariate Cox regression adjusting for age, gender, and pathological stage was used to estimate HR and 95% CI. A prognostic nomogram was developed and validated using calibration curves with bias-corrected bootstrap (B = 200 iterations).

### 4.4. In Vitro Transcriptome Profiling and Functional Enrichment

Differential gene expression was analyzed using the limma-voom pipeline (TMM normalization; DEGs defined as FDR < 0.05 and |log_2_FC| > 1). Interferon-stimulated and viral defense genes were filtered to minimize transfection artifacts, while cancer-relevant inflammatory mediators were retained. GO and KEGG enrichment analyses were performed with BH correction (FDR < 0.05). GSEA was conducted using hallmark and KEGG gene sets (FDR < 0.25). Key genes across TNF signaling, proliferation, and EMT categories were visualized by log_2_ fold change.

### 4.5. Single-Cell RNA Sequencing Analysis

Single-cell data were processed using Seurat (v5.0+). After quality control (200–6000 genes/cell, <20% mitochondrial content, >500 UMIs), 27,402 cells were retained. Data were normalized, scaled, and dimensionality-reduced by PCA and UMAP. Cells were clustered using the Louvain algorithm and annotated into 10 major cell types using curated marker gene sets. For AQP1-TNF pathway analysis, cell type-specific expression of 40 core TNF pathway genes was examined, and Pearson correlations between AQP1 and each TNF gene were computed across all cells (BH-corrected FDR < 0.05).

### 4.6. Software and Statistical Tools

All analyses were performed in R (v4.5.2) using the following packages: Seurat (v5.0+) for single-cell analysis; survival and survminer for survival analysis; limma for differential expression; clusterProfiler for functional enrichment; timeROC and pROC for ROC analyses; rms for nomogram development; and ggplot2, ggpubr, pheatmap, and patchwork for visualization. Statistical significance was set at two-sided α = 0.05, with Benjamini–Hochberg FDR < 0.05 for multiple testing correction where applicable.

### 4.7. Cell Culture and Lentiviral Transduction

RENCA cells (RRID:CVCL_2174) were provided by Shanghai Jinyuan Biotechnology Co., Ltd. (Shanghai, China) and used at the third passage (P3). The cell line was authenticated by mouse short tandem repeat (STR) profiling (18 loci; 95.52% match to the reference RenCa profile in the ExPASy database; Genetic Testing Biotechnology, Suzhou, China; report dated August 2025), and the STR report is provided as Supplementary Material. RENCA cells were cultured in RPMI-1640 medium (Gibco, Grand Island, NY, USA; Cat# 11875093) supplemented with 10% fetal bovine serum (FBS; Gibco, Grand Island, NY, USA; Cat# 10091-148) and 1% penicillin–streptomycin (Gibco, Grand Island, NY, USA; Cat# 15140122) at 37 °C with 5% CO_2_. The full-length coding sequence of mouse Aqp1 (NM_007472.2; 810 bp) was cloned into a lentiviral overexpression vector (GeneChem, Shanghai, China) in which transgene expression was driven by the ubiquitin C (UbC) promoter; the vector carried a puromycin-resistance cassette for selection and contained no fluorescent reporter. The negative control (oe-NC) was the corresponding empty vector lacking the AQP1 insert, controlling for the viral backbone, transcriptional burden, and selection independent of the transgene. RENCA cells were transduced at a multiplicity of infection (MOI) of 20, and stably transduced cells were selected with puromycin (Beyotime, Shanghai, China; Cat# ST551) at 2 μg/mL for two weeks; AQP1 overexpression was confirmed by qPCR before downstream assays.

### 4.8. RNA Extraction and Quantitative Real-Time PCR (qPCR)

Total RNA was extracted from RENCA cells using TRIzol reagent (Invitrogen, Carlsbad, CA, USA; Cat# 15596018). The concentration and purity of RNA were determined using a NanoDrop spectrophotometer (Model 1011U; Thermo Fisher Scientific, Waltham, MA, USA). cDNA was synthesized from 1 μg of total RNA using the Evo M-MLV RT Kit for qPCR (Accurate Biotechnology, Changsha, China; Cat# AG11707) with both oligo(dT)_18_ and random hexamer primers supplied with the kit, according to the manufacturer’s instructions. Quantitative real-time PCR was performed using SYBR Green PCR Master Mix (Applied Biosystems, Foster City, CA, USA; Cat# 4367659) on an ABI 7500 system (Applied Biosystems, Foster City, CA, USA). The relative mRNA expression of AQP1 was calculated using the 2^−ΔΔCt^ method, with GAPDH as the internal control.

### 4.9. Cell Proliferation Assay (CCK-8)

Cell proliferation was evaluated using the Cell Counting Kit-8 (CCK-8; Yeasen, Shanghai, China; Cat# 40203ES). The stably transduced oe-AQP1 and oe-NC RENCA cells were seeded into 96-well plates at a density of 3 × 10^3^ cells per well. At 0, 24, and 48 h after seeding, 10 μL of CCK-8 reagent was added to each well and incubated for 2 h at 37 °C. The absorbance at 450 nm was measured using a microplate reader (Multiskan FC; Thermo Fisher Scientific, Waltham, MA, USA). All experiments were performed in three independent biological replicates (n = 3).

### 4.10. Cell Viability Assay (Calcein-AM/PI Staining)

Cell viability was assessed using a Calcein-AM/PI Double Staining Kit (Yeasen, Shanghai, China; Cat# 40747ES) following the manufacturer’s instructions. Cells were stained with Calcein-AM (live, green) and Propidium Iodide (dead, red) and imaged by fluorescence microscopy (BX63; Olympus, Tokyo, Japan). Live cell rate was calculated as the percentage of Calcein-AM-positive cells among total cells. Data were obtained from three independent experiments (n = 3).

### 4.11. Scratch Assay

Oe-AQP1 and oe-NC RENCA cells were grown to ~90% confluence in 6-well plates, and a straight scratch was made in each well using a 200 μL pipette tip held perpendicular to the plate. After washing twice with PBS to remove detached cells, the cells were cultured in serum-free medium, and the same scratch region was imaged at 0, 24, and 48 h. The wound area was measured with ImageJ2 (v2.14.0; National Institutes of Health, Bethesda, MD, USA), and the scratch healing rate was calculated as (A_0_ − A_t_)/A_0_ × 100%. Data from three independent experiments are presented as the mean ± SD and compared by two-tailed unpaired Student’s *t*-test.

### 4.12. Transwell Invasion Assay

The invasive ability of RENCA cells was evaluated using Transwell inserts (8 μm pore size; Corning, NY, USA) coated with Matrigel (Corning, NY, USA). Cells (5 × 104) suspended in 200 μL of serum-free medium were seeded into the upper chamber, while 600 μL of medium containing 10% FBS was added to the lower chamber as a chemoattractant. After 24 h of incubation, cells on the upper surface were removed, and cells that invaded the lower surface were fixed with 4% paraformaldehyde (Beyotime, Shanghai, China; Cat# P0099 ) and stained with 0.1% crystal violet (Beyotime, Shanghai, China; Cat# C0121). Data were obtained from three independent experiments (n = 3).

### 4.13. Western Blotting

RENCA cell proteins were extracted using RIPA lysis buffer (Beyotime, Shanghai, China; Cat# P0013C) with protease and phosphatase inhibitors (Beyotime, Shanghai, China; Cat# P1045). Protein concentrations were determined with a BCA Protein Assay Kit (Pierce/Thermo Fisher Scientific, Rockford, IL, USA; Cat# 23225). Equal protein (30 μg) was separated using 10–12% SDS-PAGE and transferred to PVDF membranes (Merck Millipore, Burlington, MA, USA; Cat# ISEQ00010). After blocking with 5% non-fat milk(Beyotime, Shanghai, China; Cat#P0216), the membranes were incubated overnight at 4 °C with the following primary antibodies: anti-AQP1 (ab300464, Abcam, Cambridge, UK, 1:1000), anti-TNF-α (ab6671, Abcam, Cambridge, UK, 1:1000), anti-Bax (ab182733, Abcam, Cambridge, UK, 1:1000), anti-Cleaved Caspase-3 (AB3623, Merck Millipore, 1:1000), anti-TNFRSF1A (ab223352, Abcam, Cambridge, UK, 1:1000), anti-Vimentin (ab45939, Abcam, Cambridge, UK, 1:1000), and anti-β-Actin (ab8227, Abcam, Cambridge, UK, 1:5000). The membranes were then incubated with HRP-conjugated goat anti-rabbit IgG H&L secondary antibody (ab6721, Abcam, Cambridge, UK, 1:10,000) for 1 h at room temperature. Bands were visualized by ECL (Millipore, Burlington, MA, USA; Cat#WBKLS0500), normalized to β-Actin, and quantified with ImageJ.

### 4.14. Tumor Xenograft Model in Nude Mice

All animal experiments were approved by the Institutional Animal Care and Use Committee of Dalian Medical University (protocol code XL250716173). Four-week-old male BALB/c nude mice (BALB/c-nu/nu, athymic) were obtained from Shandong Pengyue Laboratory Animal Technology Co., Ltd. (Jinan, China) and randomly assigned into two groups (n = 5 per group). RENCA cells stably expressing AQP1 or NC (5 × 10^6^ cells in 100 μL PBS) were subcutaneously injected into the right flank of the mice. Tumor volume (V) was measured every 7 days using the modified ellipsoid formula V = (length × width^2^)/2 [47]. After 30 days, mice were sacrificed, and tumors were excised, photographed, and weighed. Tumor tissues were then fixed in 4% paraformaldehyde for histological and immunohistochemical analysis.

### 4.15. Histological and Immunohistochemical (IHC) Analysis

Paraffin-embedded tumor tissues were sectioned at 4 μm. Sections were stained with HE for morphological assessment. For IHC, sections were deparaffinized, rehydrated, and subjected to antigen retrieval, followed by blocking and overnight incubation with primary antibodies against AQP1 (ab300464, Abcam, Cambridge, UK, 1:100) and Cleaved Caspase-3 (AB3623, Merck Millipore, 1:100) at 4 °C. Signal was detected using a DAB substrate kit (Yeasen, Shanghai, China; Cat# 36302ES01) and sections were counterstained with hematoxylin (Beyotime, Shanghai, China; Cat# C0107).

## 5. Limitations

Several limitations should be acknowledged. First, this study relied on retrospective TCGA data; prospective validation in independent multi-center cohorts is needed before clinical implementation. Second, our in vitro and in vivo experiments used mouse RENCA cells; validation in human ccRCC cell lines and patient-derived models would strengthen translational relevance. Third, because oe-AQP1 cells proliferate more slowly in vitro, the reduced xenograft growth is consistent with, but does not independently prove, a specific apoptosis-driven mechanism, as the in vitro and in vivo growth deficits cannot be fully disentangled. Fourth, a parental, non-transduced RENCA xenograft arm was not included; although the empty-vector (oe-NC) control accounts for backbone-, transduction-, and selection-related effects, a native baseline would further exclude any vector-intrinsic influence on tumorigenicity. Fifth, the association between AQP1, TNF signaling, and apoptosis in vivo is correlative, being based on Western blot, HE, and immunohistochemical analyses; direct functional validation—for example, TNF/TNFR1 blockade or genetic perturbation combined with in situ apoptosis assays—is required to establish causality. Sixth, the single-cell dataset was from limited patients; larger atlases would better capture tumor heterogeneity. Finally, the modest methylation-expression correlation suggests that DNA methylation is one of multiple regulatory mechanisms warranting further investigation. Although our TCGA analyses support an association between AQP1 promoter hypermethylation and transcriptional silencing in ccRCC, this was not experimentally confirmed at the locus level; direct validation by methylation-specific PCR or bisulfite sequencing in ccRCC cell lines and tissues is warranted in future studies.

## Figures and Tables

**Table 1:** Tumor volume and weight in the xenograft model at day 30 (mean ± SD, n = 5 per group).

Parameter	oe-NC	oe-AQP1	*p*-Value
Tumor volume (mm^3^)	522.81 ± 96.45	212.88 ± 46.55	<0.001
Tumor weight (g)	0.378 ± 0.058	0.138 ± 0.034	<0.001

**Table 2:** Sample Sizes and Cohort Characteristics.

Dataset	Sample Size	Description
TCGA-KIRC	610	538 tumor, 72 normal (72 paired)
Stage I	271	50.6% of staged tumors
Stage II	58	10.8% of staged tumors
Stage III	123	22.9% of staged tumors
Stage IV	84	15.7% of staged tumors
Survival cohort	532	Tumors with complete clinical data
In vitro RNA-seq	6	3 AQP1-overexpression, 3 controls
scRNA-seq (GSE159115)	27,402	Post-QC cells, 10 annotated cell types

## Data Availability

Publicly available datasets were analyzed in this study. TCGA-KIRC data can be found at the GDC Data Portal (https://portal.gdc.cancer.gov/). Single-cell RNA sequencing data are available in GEO under accession number GSE159115. The raw RNA-seq data generated in this study have been deposited in GEO under accession number GSE327897 and will be publicly released on 31 May 2026.

## Supplementary Materials

The following supporting information can be downloaded at: https://www.mdpi.com/article/10.3390/ijms27125215/s1.

## Abbreviations

The following abbreviations are used in this manuscript:

AQP1	Aquaporin-1
BCA	Bicinchoninic acid assay
BH	Benjamini–Hochberg
CCK-8	Cell Counting Kit-8
ccRCC	Clear cell renal cell carcinoma
DNMTi	DNA methyltransferase inhibitors
EMT	Epithelial-mesenchymal transition
ERVs	Endogenous retroviruses
HE	Hematoxylin and Eosin
HR	Hazard ratio
ICB	Immune checkpoint blockade
ICI	Immune checkpoint inhibitors
MOI	Multiplicity of infection
NES	Normalized enrichment score
NF-κB	Nuclear factor kappa-light-chain-enhancer of activated B cells
TNFRSF1A	TNF receptor superfamily member 1A
